# Beyond Obesity: Sex-Specific Associations of Vaspin with Metabolic Dysfunction

**DOI:** 10.3390/jcm15166284

**Published:** 2026-08-13

**Authors:** Marcelina Sperling, Krystyna Czyżewska, Marta Pelczyńska, Paweł Bogdański, Dorota Formanowicz, Edyta Mądry, Teresa Grzelak

**Affiliations:** 1 Chair and Department of Medical Chemistry and Laboratory Medicine, Poznan University of Medical Sciences, 8 Rokietnicka Street, 61-701 Poznan, Poland; doforman@ump.edu.pl; 2 Department of Nursing, Stanislaw Staszic State University of Applied Sciences in Pila, 10 Podchorazych Street, 64-920 Pila, Poland; kczyzewska@ans.pila.pl; 3 Department of Treatment of Obesity, Metabolic Disorders and Clinical Dietetics, Poznan University of Medical Sciences, 49 Przybyszewskiego Street, 60-355 Poznan, Poland; mpelczynska@ump.edu.pl (M.P.); pbogdanski@ump.edu.pl (P.B.); 4 Physiology Department, Poznan University of Medical Sciences, 11 Smoluchowskiego Street, 60-179 Poznan, Poland; emadry@ump.edu.pl

**Keywords:** vaspin, SERPINA12, adipokines, batokines, total antioxidant status, metabolic syndrome, insulin resistance, sex differences, visceral obesity

## Abstract

**Background:** Vaspin (SERPINA12) is an adipokine implicated in the regulation of insulin sensitivity and metabolic homeostasis. Evidence regarding its association with obesity-related metabolic disturbances remains inconsistent, and potential sex-specific differences are not fully understood. **Methods:** This cross-sectional study included 81 adults aged 30–60 years (38 women and 43 men), divided into a metabolic syndrome group (MS+) and a control group (MS−). Anthropometric, biochemical, total antioxidant status and blood pressure parameters were assessed, and insulin resistance was estimated using the HOMA-IR index. **Results:** In MS+ men, serum vaspin concentrations were higher than in MS− men (*p* = 0.004) and were associated with BMI, waist circumference, body fat percentage, fasting insulin, HOMA-IR, and diastolic blood pressure (*p* < 0.05 for all). In multivariable regression analysis (adjusted for age), insulin levels, lean body mass, and diastolic blood pressure explained up to 15.2% of the variability in serum vaspin concentrations in men (*p* = 0.035), whereas no significant model was observed in women. ROC (receiver operating characteristic) curve analysis demonstrated better discriminative performance of serum vaspin for identifying metabolic syndrome in men than in women (AUC: 0.784 ± 0.094 vs. 0.416 ± 0.115, respectively; *p* = 0.013). **Conclusions:** Circulating vaspin concentrations are more strongly associated with metabolic disturbances in men than in women These findings suggest that sex-specific regulation of vaspin may contribute to differences in metabolic dysfunction associated with visceral obesity. Overall, these results indicate that vaspin may reflect visceral obesity-related metabolic dysfunction rather than general adiposity. Further studies are needed to clarify the mechanistic role of vaspin and to determine whether it has potential as a biomarker of metabolic dysfunction.

## 1. Introduction

Obesity and metabolic syndrome are among the most significant contemporary global public health challenges and are strongly associated with insulin resistance, type 2 diabetes, dyslipidemia, arterial hypertension, and increased cardiovascular risk [1,2]. Adipose tissue is currently regarded not only as an energy storage organ but also as an active endocrine organ secreting numerous bioactive molecules, including adipokines, which regulate glucose and lipid metabolism, inflammatory responses, vascular homeostasis, and insulin sensitivity. Dysregulation of adipokine secretion substantially contributes to the development and progression of obesity-related metabolic disorders [1,2,3,4,5].

Vaspin (visceral adipose tissue-derived serine protease inhibitor), also known as SERPINA12, is a relatively recently discovered adipokine first isolated from the visceral adipose tissue of Otsuka Long-Evans Tokushima Fatty (OLETF) rats, a well-established animal model of obesity and type 2 diabetes [6,7]. Vaspin has emerged as an important adipokine involved in the pathophysiology of metabolic syndrome. Experimental and clinical studies suggest that it may exert protective metabolic effects by improving insulin sensitivity and attenuating obesity-associated chronic inflammation. Furthermore, vaspin has been shown to exert beneficial effects on endothelial cells and vascular smooth muscle cells under hyperglycemic and hyperlipidemic conditions. Accumulating evidence also indicates that vaspin may be associated with a reduced risk of atherosclerosis and ischemic stroke [8,9,10,11].

Despite the growing number of studies investigating the role of vaspin in metabolic disorders, substantial discrepancies remain between findings from animal and in vitro studies and those obtained in human populations. Several studies have demonstrated positive associations between circulating vaspin levels and obesity, visceral adipose tissue accumulation, insulin resistance, metabolic syndrome, and type 2 diabetes [12,13,14,15]. Elevated vaspin concentrations have been interpreted as a compensatory mechanism in response to worsening metabolic status and progressive insulin resistance [7,12,13,16] and, according to more recent reports, as a predictor of type 2 diabetes (T2D) risk [17].

An important factor that may influence circulating vaspin levels is biological sex. Women and men differ significantly in adipose tissue distribution, hormonal profiles, inflammatory profiles, and insulin sensitivity. Visceral adipose tissue, which is generally more abundant in men, is characterized by higher metabolic activity and a stronger association with cardiometabolic risk than subcutaneous adipose tissue.

Although sexual dimorphism in circulating vaspin levels has been described, the influence of biological sex on the relationship between vaspin concentrations and metabolic disturbances remains incompletely understood. Previous studies have yielded inconsistent results regarding the association between circulating vaspin levels and individual components of the metabolic syndrome, and observed sex-related differences have not been consistent across populations. Therefore, further studies evaluating these relationships separately in women and men are warranted [18].

The aim of the present study was to assess sex-dependent associations between serum vaspin concentrations and selected anthropometric and biochemical parameters, as well as blood pressure values in adults with and without metabolic syndrome.

## 2. Materials and Methods

### 2.1. Study Design

This cross-sectional study was designed to evaluate the association between circulating vaspin concentrations and metabolic syndrome components, with particular emphasis on sex-specific differences.

#### 2.1.1. Study Population

The study population consisted of 81 individuals aged 30–60 years, including 38 women (median age, 45 years; Q1–Q3: 39–54 years) and 43 men (median age, 43 years; Q1–Q3: 39–50 years). Age distribution did not differ significantly between women and men. The metabolic syndrome group (MS+) consisted of 60 individuals (32 men and 28 women) who were patients attending the Department of Treatment of Obesity, Metabolic Disorders and Clinical Dietetics at Poznan University of Medical Sciences. The control group (MS−) comprised 21 participants (11 men and 10 women), recruited through public advertisements. Inclusion criteria for the control group were as follows: age 30–60 years, BMI between 18.5 and <30 kg/m^2^, and waist circumference < 88 cm in women and <102 cm in men (Table 1). Participants were assigned to the study and control groups based on evaluations of available medical records and detailed medical and nutritional interviews. The assessment included both current and previous medical conditions, medication and dietary supplement use, dietary habits, and levels of physical activity.

#### 2.1.2. Inclusion and Exclusion Criteria

The study group consisted of individuals diagnosed with metabolic syndrome (MS+), whereas the control group included participants without metabolic syndrome (MS−). Metabolic syndrome was diagnosed according to International Diabetes Federation (IDF), American Heart Association (AHA), National Heart, Lung, and Blood Institute (NHLBI). According to this definition, metabolic syndrome is diagnosed when at least three of the following five criteria are present:

1. Abdominal obesity (increased waist circumference)—waist circumference thresholds are ethnicity-specific. For the Caucasian population: men: ≥94 cm or ≥102 cm; women: ≥80 cm or ≥88 cm.
2. Hypertriglyceridemia—triglyceride concentration ≥ 150 mg/dL (1.7 mmol/L) or specific treatment for elevated triglycerides.
3. Reduced HDL cholesterol: men: <40 mg/dL (1.03 mmol/L), women: <50 mg/dL (1.29 mmol/L), or specific treatment for reduced HDL cholesterol.
4. Elevated blood pressure—systolic blood pressure ≥ 130 mmHg and/or diastolic blood pressure ≥ 85 mmHg, or current antihypertensive drug treatment.
5. Elevated fasting plasma glucose—fasting plasma glucose ≥ 100 mg/dL (5.6 mmol/L) or previously diagnosed type 2 diabetes mellitus.

**Figure 1:**
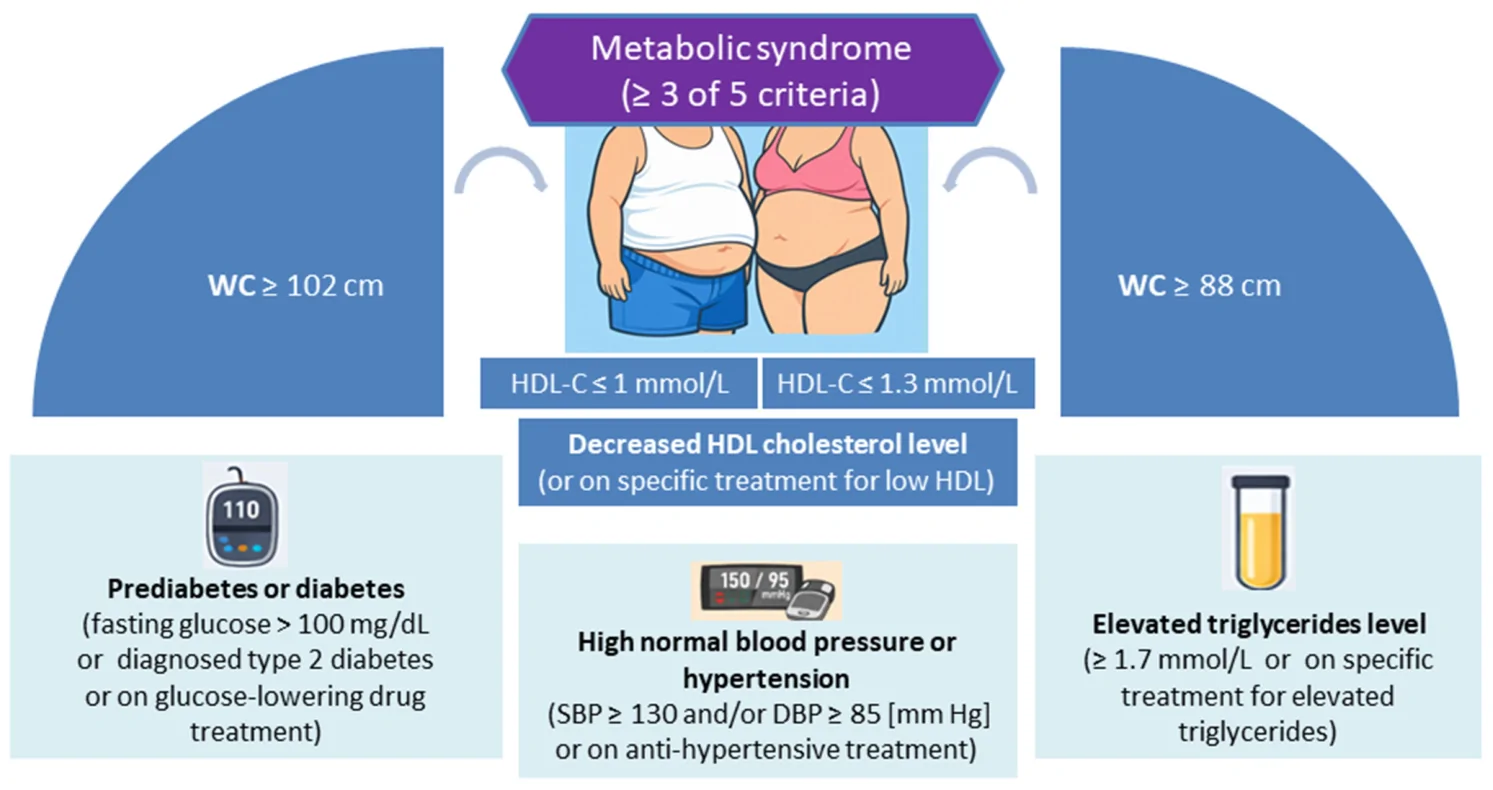
Source: prepared by the authors based on [19]. WC—waist circumference, SBP—systolic blood pressure, DBP—diastolic blood pressure, HDL-C—High-Density Lipoprotein Cholesterol.

Although an increased risk of cardiovascular disease and diabetes is observed at waist circumferences of ≥94 cm in men and ≥80 cm in women of Caucasian ethnicity, the risk is substantially higher at waist circumferences of ≥102 cm in men and ≥88 cm in women. Therefore, only Caucasian participants meeting these higher waist circumference thresholds ≥ 102 cm for men and ≥88 cm for women) were included in the study to ensure the inclusion of individuals with more pronounced abdominal obesity and a higher metabolic risk [19]. Details are presented in Figure 1.

Exclusion criteria included chronic disorders of the endocrine glands, liver, kidneys, or pancreas, as well as a history of cancer or cardiovascular events within the five years preceding enrolment. Participants previously diagnosed with dyslipidemia and/or hypertension continued their prescribed pharmacotherapy throughout the study period. None of the participants had been previously diagnosed with or treated for diabetes mellitus or impaired glucose tolerance.

Only individuals who, on the day of assessment, did not report malaise or symptoms suggesting acute infection were included in the analyses. According to participants’ declarations, body weight remained stable during the three months prior to enrolment. Physical activity was assessed by self-report during the clinical interview. None of the participants were professional athletes, and all reported low to moderate habitual levels of physical activity. Shift workers were excluded from the study. Nutritional habits were evaluated using 3-day dietary records, which allowed the exclusion of participants following non-standard dietary patterns or alternative diets, such as vegetarian or ketogenic diets, that could potentially influence circulating vaspin concentrations. Pregnancy and lactation, whether current or within the previous six months, constituted additional exclusion criteria.

Patients with metabolic syndrome were recruited from a hospital setting, whereas control participants were volunteers recruited through a public advertisement. Although all participants underwent the same standardized clinical, laboratory, anthropometric, and dietary assessments, the use of different recruitment strategies may have introduced selection bias and should be considered when interpreting the findings.

### 2.2. Data Collection and Measurements

#### 2.2.1. Anthropometric Measurements

Anthropometric assessments were performed in the morning after an overnight fast. All measurements were obtained with participants wearing light underwear. Prior to examination, participants were instructed to refrain from alcohol, caffeinated beverages, tea, or energy drinks for at least 24 h and to avoid vigorous physical activity. Body weight and height were measured using a certified electronic scale (SECA 285 Wireless, Hamburg, Germany). Waist circumference was measured midway between the lower rib margin and the iliac crest, whereas hip circumference was assessed at the level of the greater trochanters, with an accuracy of 0.5 cm. These measurements were used to calculate BMI and waist-to-hip ratio (WHR).

Body composition was evaluated using bioelectrical impedance analysis (BIA) with a Bodystat 1500 analyzer (Bodystat Ltd., London, UK). The procedure was conducted in the supine position according to the manufacturer’s recommendations and included input of data on sex, height, body weight, and physical activity level. The BIA technique measures tissue resistance to a low-intensity electrical current (≤0.1 mA). Four electrodes were attached to the hands and feet, enabling assessment of body fat percentage, lean body mass, total muscle mass, and total body water content.

#### 2.2.2. Blood Pressure Assessment

Prior to blood pressure evaluation, all participants remained seated at rest for at least 5 min. Systolic and diastolic blood pressure values were measured three times at 2 min intervals using a validated automatic sphygmomanometer (Omron 705IT, Omron Corporation, Kyoto, Japan). The final values were calculated as the mean of the three measurements, in accordance with the guidelines of the European Society of Hypertension and the European Society of Cardiology [20].

#### 2.2.3. Biochemical Analyses

Venous blood samples were obtained in the morning after an overnight fasting (8–12 h). Routine biochemical parameters, including glucose, triglycerides, total cholesterol, HDL-C, and insulin concentrations, were analyzed immediately after collection. For adipokine determination, serum samples were centrifuged and subsequently stored at −80 °C until further analysis.

Serum glucose concentration was determined enzymatically using the hexokinase method with a Cobas c analyzer (Roche Diagnostics GmbH, Mannheim, Germany). Insulin levels were measured using immunochemical assays performed according to Chemi-flex protocols (Abbott Diagnostics, Lake Forest, IL, USA). Fasting glucose and insulin concentrations were subsequently used to estimate insulin resistance using the HOMA-IR index (Homeostasis Model Assessment of Insulin Resistance) index according to the standard formula: HOMA-IR = (fasting glucose [mmol/L] × fasting insulin [µU/L])/22.5.

Serum triglycerides, total cholesterol, and HDL-C (High-Density Lipoprotein Cholesterol) concentrations were determined using commercially available enzymatic colorimetric assays. Since triglyceride concentrations in all participants remained below 400 mg/dL (4.5 mmol/L), LDL-C (Low-Density Lipoprotein Cholesterol) levels were calculated using the Friedewald equation [21].

The total antioxidant status (TAS) in plasma was determined using a colorimetric assay (Randox Laboratories Ltd., Crumlin, UK). In this method, samples are incubated with the chromogenic substrate ABTS, followed by peroxidase (metmyoglobin) and hydrogen peroxide, generating ABTS^+^ radical cations measured at 600 nm with an MR 96 microplate reader (CLINDIAG SYSTEMS B.V.B.A., Pollare, Belgium). Results are expressed as Trolox equivalents (mmol/L), reflecting antioxidant capacity relative to the water soluble vitamin E analog. Analytical performance showed intra and inter assay coefficients of variation up to 5.0%.

Serum vaspin concentrations were assessed using a certified enzyme immunoassay kit (Vaspin (human) EIA Kit, Cayman Chemical Company, Ann Arbor, MI, USA) according to the manufacturer’s instructions. Absorbance measurements were performed using an MR-96 microplate reader (CLINDIAG SYSTEMS B.V.B.A., Pollare, Belgium). Based on the obtained absorbance values, a four-parameter logistic standard calibration curve was generated in SigmaPlot 11.00, enabling the quantitative determination of serum vaspin concentrations. Intra-assay and inter-assay coefficients of variation were 11.0% and 12.1%, respectively.

### 2.3. Ethical Approval and Informed Consent

The study was conducted according to the guidelines of the Declaration of Helsinki, and approved by The Local Bioethics Committee of Poznan University of Medical Sciences (protocol no. 805/15). Written informed consent to participate in the study was obtained from all participants.

### 2.4. Statistical Analyses

Statistical analyses were performed using STATISTICA 14 (StatSoft Inc., Tulsa, OK, USA) and PQStat version 1.8.6 (Plewiska, Poland). The normality of data distribution was assessed using the Shapiro–Wilk test. For quantitative variables meeting the assumptions of normality and homogeneity of variance, as verified by Levene’s test, one-way ANOVA for independent samples was applied. Variables that did not meet the assumption of normal distribution were analyzed using the nonparametric Mann–Whitney U test. In cases of unequal variances despite normal distribution, Welch’s test was used.

Correlations between variables were evaluated using Spearman’s rank correlation coefficient for data that did not fulfil the assumption of normality. Correlation coefficients (R_s_) were interpreted as strong when R_s_ > 0.6, moderate when R_s_ ranged from 0.4 to 0.6, and weak when R_s_ < 0.4, provided that the *p*-value was <0.05. Analogous criteria were applied to negative correlations, with negative R_s_ values indicating inverse relationships between variables [22].

The influence of selected variables on serum vaspin concentrations was assessed using multivariable regression analysis. Additionally, receiver operating characteristic (ROC) curves and the corresponding area under the curve (AUC) were used to evaluate the discriminative ability of vaspin in the male and female populations for distinguishing individuals with and without metabolic syndrome. AUC values range from 0 to 1, with higher values indicating better discriminatory performance [23]. The formulas used for calculating sensitivity, specificity, and 95% confidence intervals of AUC are presented in Table S1. Statistical significance was set at *p* < 0.05 for all analyses. Bonferroni corrections were applied whenever multiple pairwise comparisons were performed to control the family-wise type I error rate and minimize the probability of false-positive findings across tested contrasts.

Sample size calculations were performed assuming a statistical power of 0.80 and a type II error probability (β) of 0.20 to determine the minimum sample size required to detect statistically significant differences between the study groups [24]. Mean vaspin concentrations and standard deviations (SD) were derived from published data and from laboratory ranges established in our preliminary measurements. A statistical power of 0.80 (β = 0.20) was assumed, as this value is widely accepted as the conventional minimum for adequately powered biomedical studies and provides an appropriate balance between minimizing the risk of a type II error and maintaining a feasible sample size [24,25]. Statistical significance was set at *p* < 0.05 for all analyses.

## 3. Results

### 3.1. Anthropometric, Biochemical and Blood Pressure Measurements in Analysed Population

#### 3.1.1. Anthropometric Analyses

The study involved 81 participants, including 38 women and 43 men. Although median BMI in the female population was 31.15 (24.30; 41.65) [kg/m^2^] and was similar to that in the male population 33.6 (27.00; 41.55) [kg/m^2^], men showed significantly higher values than women for body weight (*p* = 0.0001), waist circumference (*p* = 0.003), WHR (*p* < 0.0001) and body lean percentage (*p* < 0.0001) (Table 2).

#### 3.1.2. Characteristics of Selected Physical and Biochemical Parameters

In comparison with the male population, the female group had higher LDL-C (*p* = 0.013), HDL-C (*p* = 0.021) and total cholesterol (*p* = 0.020) concentrations and lower triglycerides (*p* = 0.005), fasting glucose (*p* = 0.011), and HOMA-IR values (*p* = 0.035). Moreover, fasting insulin and levels of systolic and diastolic blood pressure were similar in women and men (Table 3).

#### 3.1.3. Vaspin Concentration in Selected Subgroups

**Figure 2:**
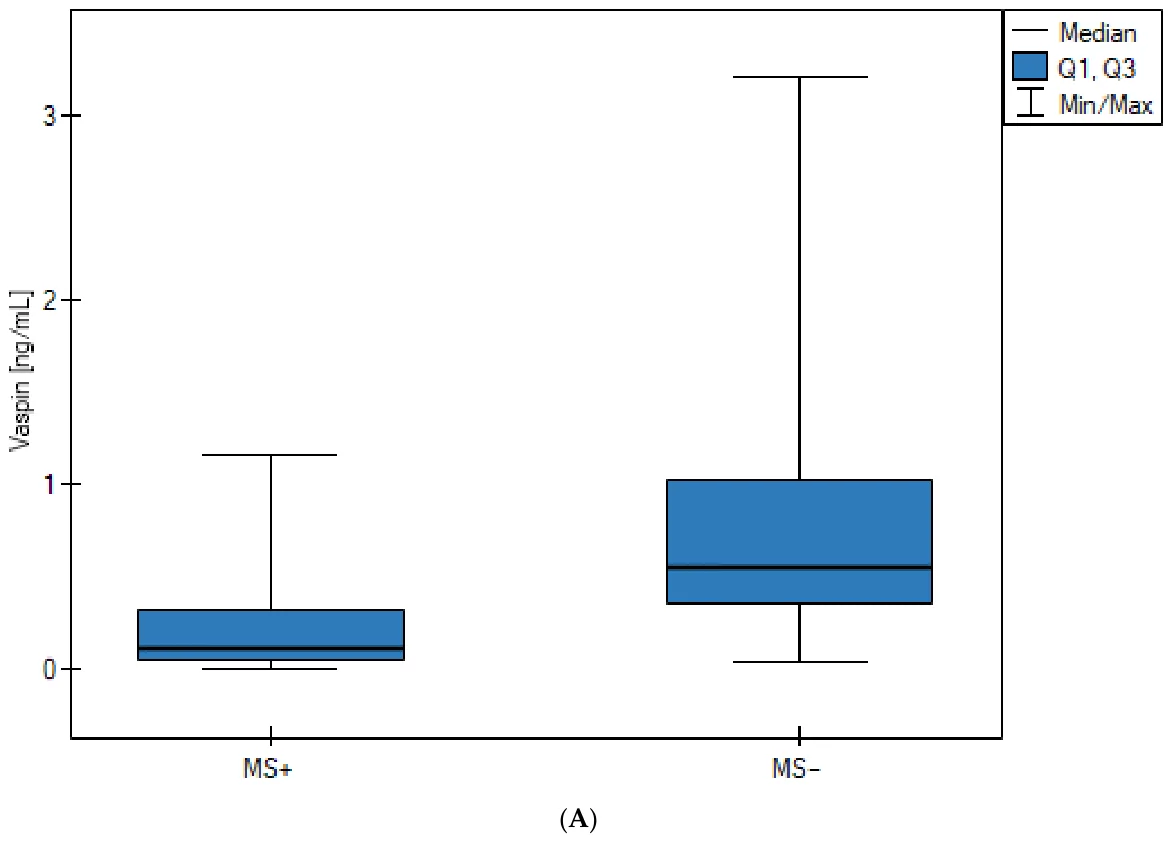

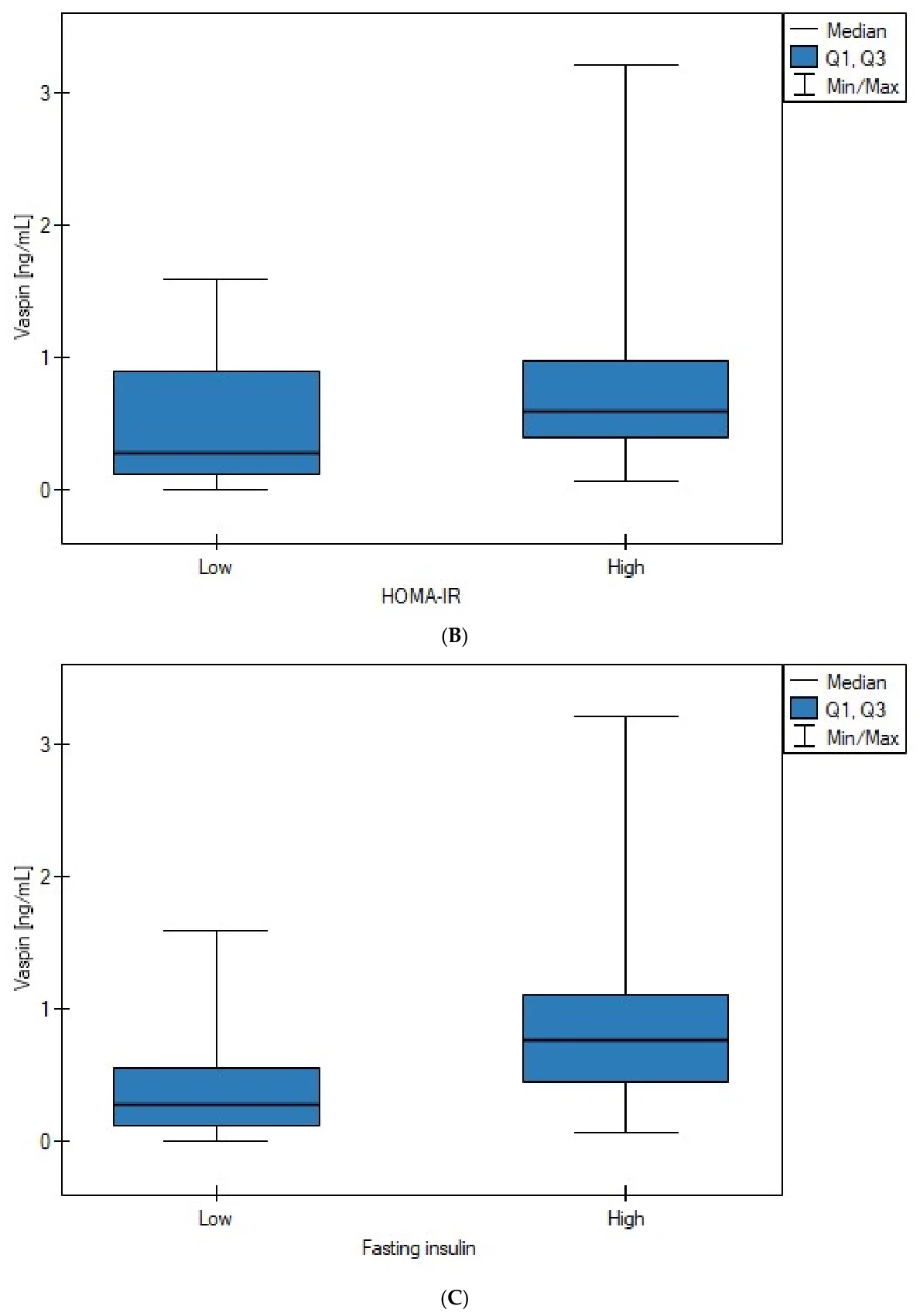
Comparison of vaspin concentration [ng/mL] in selected subgroups of the male population (*n* = 43). *n*—number of people; *p*—level of statistical significance; HOMA-IR—Homeostatic Model Assessment-Insulin Resistance; MS−—group without metabolic syndrome; MS+—group with metabolic syndrome. (**A**): in subgroup without metabolic syndrome (MS−, *n* = 11) vs. subgroup with metabolic syndrome (MS+, *n* = 32) (*p* = 0.004 in Mann–Whitney U test). (**B**): in HOMA-IR < 2.5 subgroup (*n* = 21) vs. HOMA-IR ≥ 2.5 subgroup (*n* = 22) (*p* = 0.036 in Mann–Whitney U test). (**C**): in fasting insulin < 12 µU/mL subgroup (*n* = 25) vs. fasting insulin ≥ 12 µU/mL subgroup (*n* = 18) (*p* = 0.006 in Mann–Whitney U test). The solid line indicates the median value.

Vaspin concentrations did not differ significantly between the study and control groups and were similar in the entire female and male groups (Table 3), but a higher vaspin concentration was observed in the male MS+ group compared with the male MS− (Table 4, Figure 2A). Moreover, in the male population, we observed higher vaspin concentrations in the subgroup with HOMA-IR ≥ 2.5 vs. the subgroup with lower HOMA-IR values (*p* = 0.036, Figure 2B) and in the subgroup with fasting insulin ≥ 12 µU/mL vs. the subgroup with lower levels of insulin (*p* = 0.005, Figure 2C). Similar differences were not observed in women (Table 5).

### 3.2. Correlations Between Vaspin Concentration and Selected Anthropometric, Biochemical and Physiological Parameters

#### 3.2.1. Correlations Between Vaspin Concentration and Selected Anthropometric Parameters

**Figure 3:**
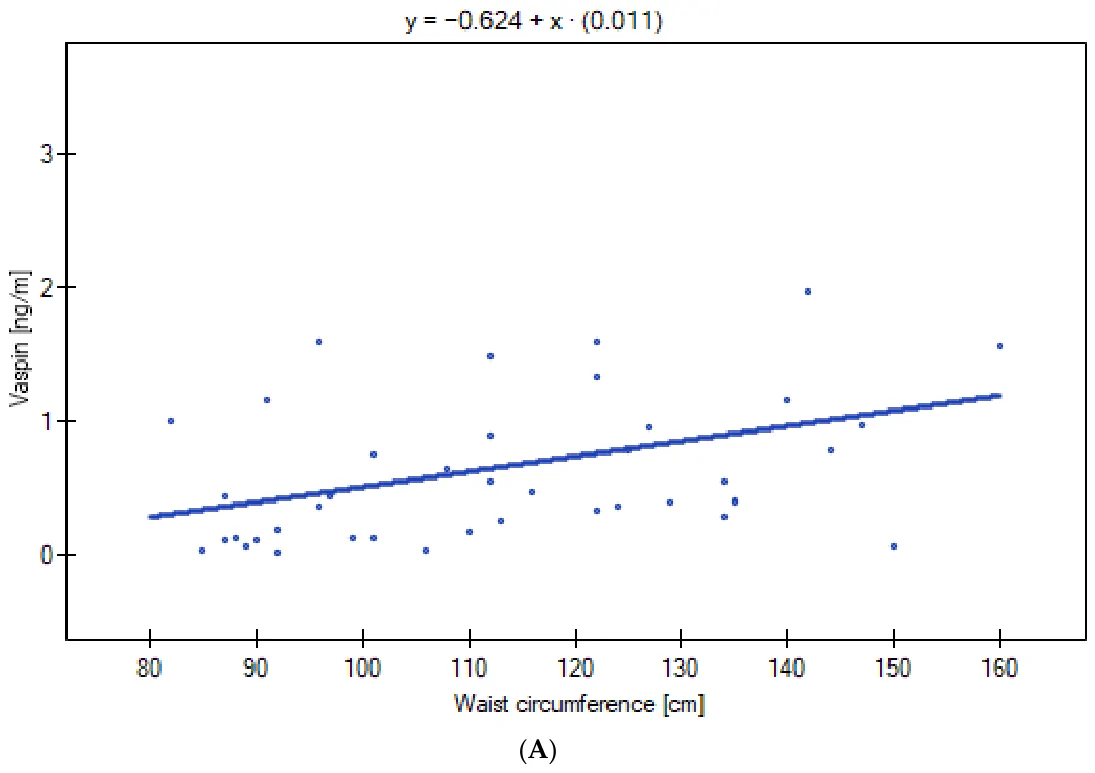

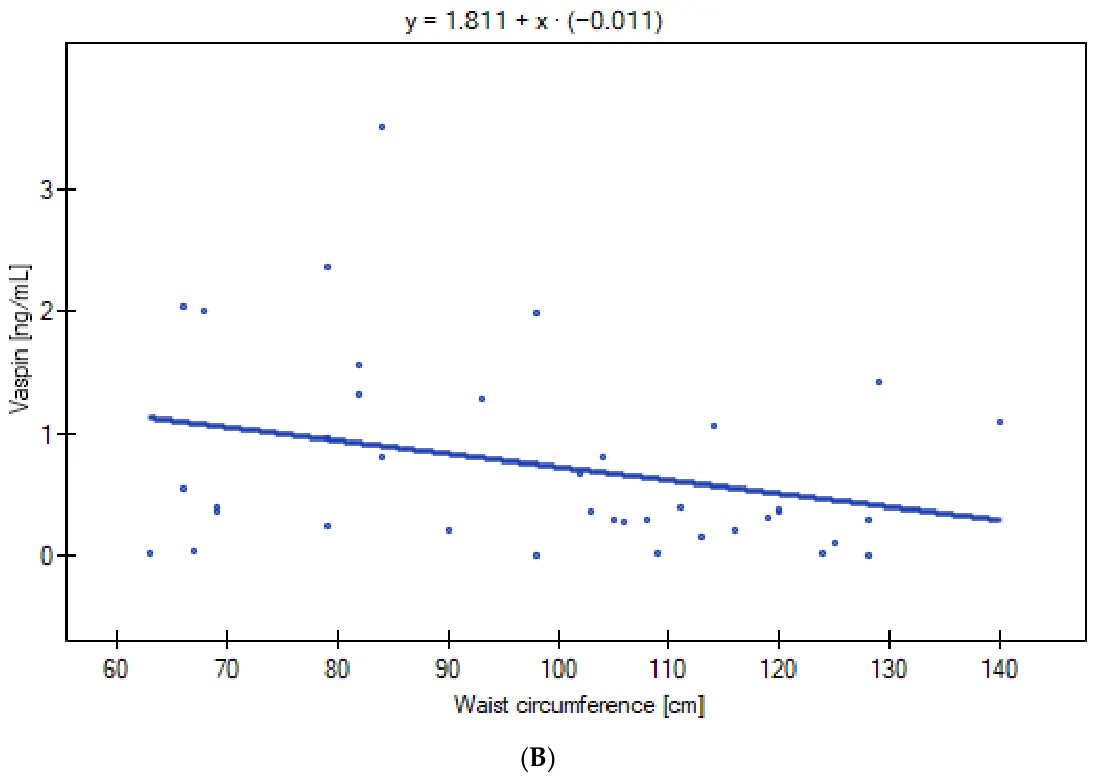
Correlation between waist circumference [cm] and vaspin concentration [ng/mL]. (**A**): Positive correlation between waist circumference and vaspin concentration in male population, *n* = 43 (R_s_ = 0.437, *p* = 0.002). (**B**): Negative correlation between waist circumference and vaspin concentration in female population, *n* = 38 (R_s_ = −0.272, *p* = 0.049).

In the male population, we observed a moderate negative correlation between vaspin concentration and body lean percentage (R_s_ = −0.423, *p* = 0.002), as well as moderate positive correlations with BMI (R_s_ = 0.426, *p* = 0.002), waist circumference (R_s_ = 0.437, *p* = 0.002), and body fat percentage (R_s_ = 0.423, *p* = 0.002). The correlation between vaspin concentration and body weight was weakly positive (R_s_ = 0.385, *p* = 0.005). Interestingly, in the female population, the direction of these associations differed from those observed in men: serum vaspin concentrations showed a weak negative correlation with waist circumference (R_s_ = −0.272, *p* = 0.049). Additionally, trends toward negative correlations were observed with WHR (R_s_ = −0.262, *p* = 0.057) and body fat percentage (R_s_ = −0.261, *p* = 0.057), together with a trend toward a positive correlation with body lean percentage (R_s_ = 0.261, *p* = 0.057) (Table 6, Figure 3A,B). For the entire population, we did not observe a correlation between vaspin concentration and analysed anthropometric parameters (Table 6).

#### 3.2.2. Correlations Between Vaspin Concentration and Selected Physiological and Biochemical Parameters

In the male population, we observed moderate positive correlations between vaspin concentration and fasting insulin (R_s_ = 0.403, *p* = 0.004) and HOMA-IR (R_s_ = 0.429, *p* = 0.002), as well as weak positive correlations with age (R_s_ = 0.302, *p* = 0.025) and diastolic blood pressure (R_s_ = 0.329, *p* = 0.016). In contrast, no significant correlations between vaspin concentration and the analysed biochemical parameters were observed in women or in the entire population (Table 7). The correlation between vaspin concentration and TAS level was weakly positive in the MS+ group (R_s_ = 0.276, *p* = 0.033). This association was not observed in the entire population or in the MS− group, regardless of sex.

### 3.3. Regression-Based Analyses

We conducted a multiple regression analysis to assess the effects of insulin concentration, body composition, and diastolic blood pressure on vaspin concentration. The multivariable regression model showed a trend toward an association between insulin concentration and lean body mass [%] with the variability of serum vaspin levels in the male population (*p* = 0.105, R^2^_adj_. (adjusted R-squared) = 7.8%) (Table 8A). To further explore this relationship, an additional variable, diastolic blood pressure (DBP), was included in the model (Table 8B). Adding DBP to the multivariable model improved the explanatory power, accounting for up to 15.2% of the variance in vaspin levels (*p* = 0.035). In the male population, the overall model was statistically significant (F = 2.88, *p* (model) = 0.035), explaining 15.2% of the variance. If insulin levels and the percentage of lean body mass remain unchanged, every 1 mmHg increase in diastolic blood pressure is associated with a 19 pg/mL higher vaspin concentration in the male population. The results demonstrate that only DBP is an independent predictor of vaspin concentration (*p* = 0.043) in the male subgroup (Table 8B). No such association was found in the women’s group (Table 8C,D) or in the case of entire population. Although statistically significant, the model’s explanatory power was moderate, suggesting that additional unmeasured factors may contribute to variability in circulating vaspin concentrations.

### 3.4. Receiver Operating Characteristic (ROC) Curve Analysis

**Figure 4:**
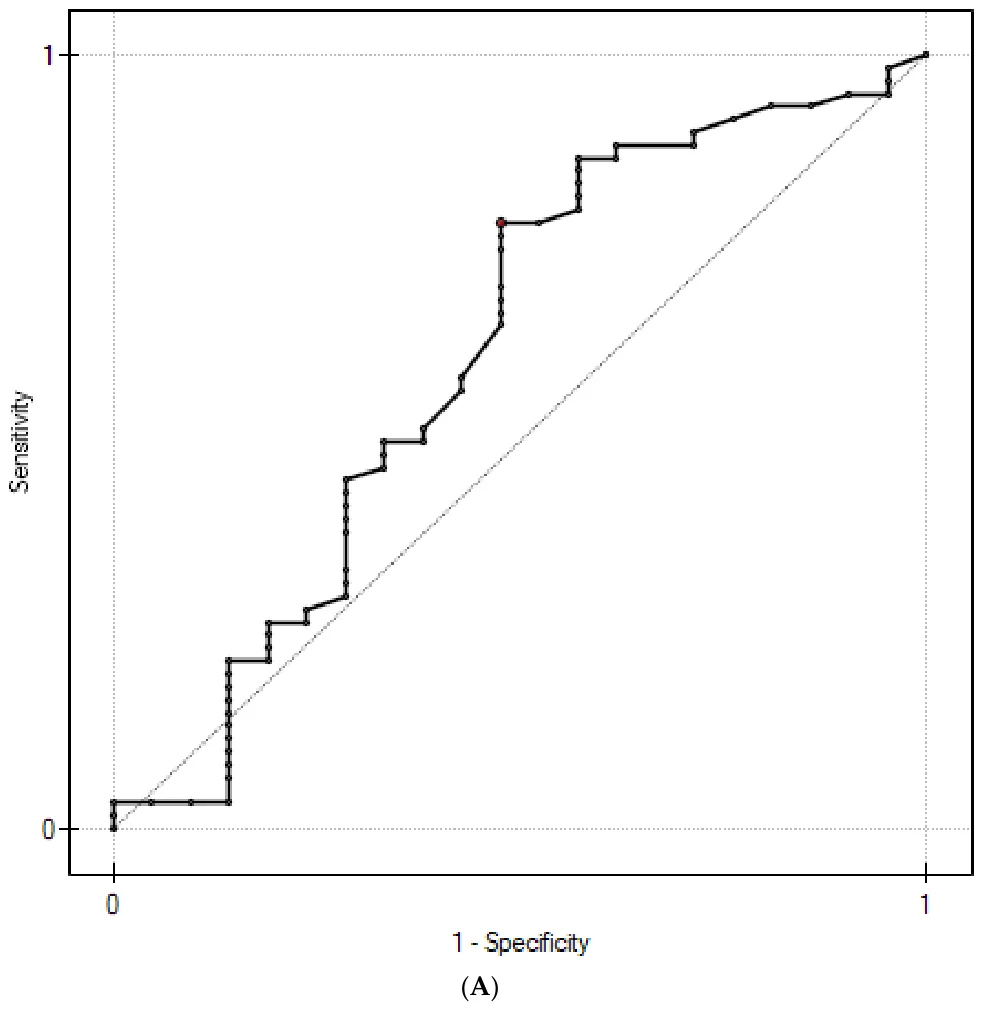

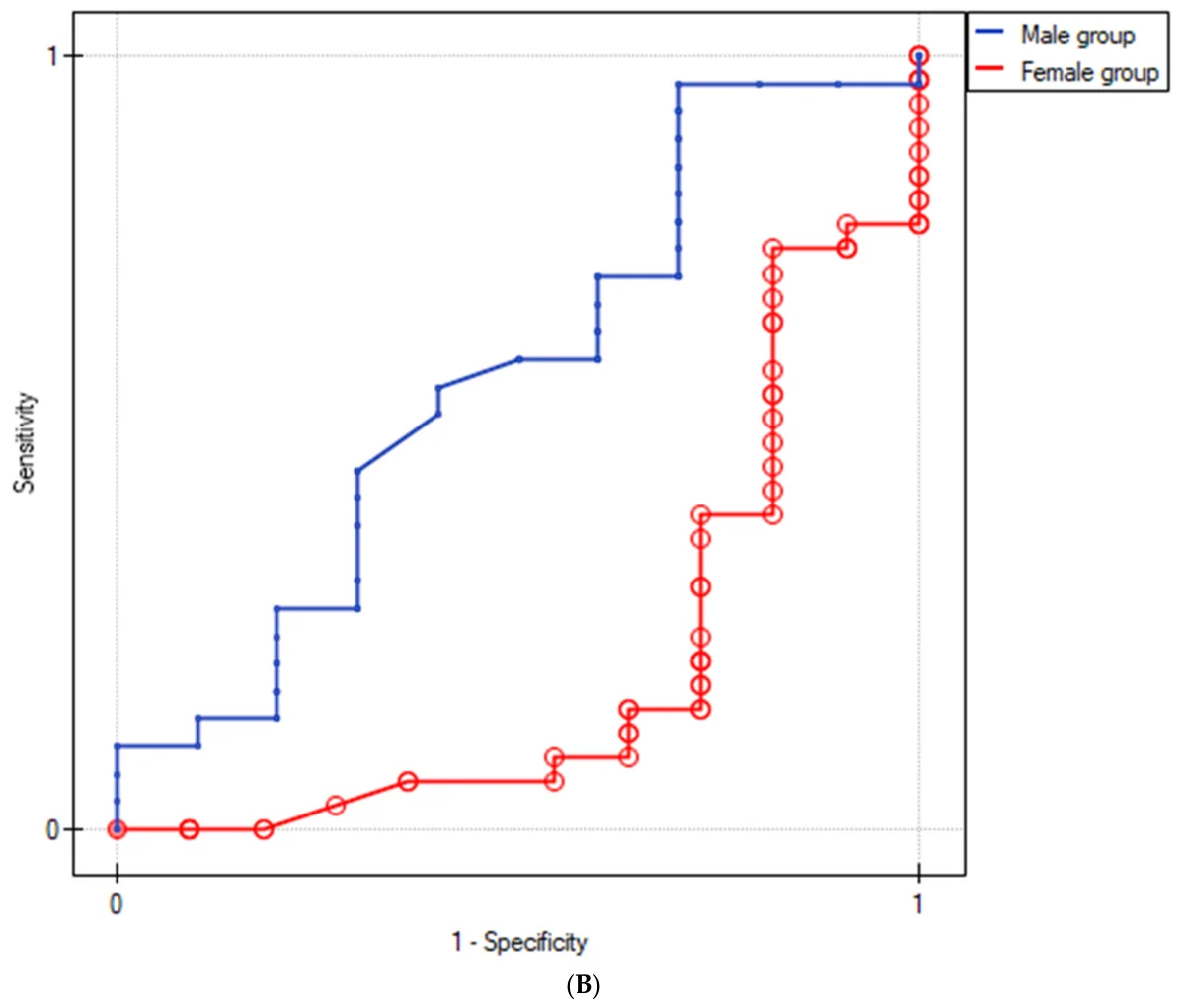
Receiver operating characteristics (ROC) curve comparison for vaspin in (**A**) the entire study group (*n* = 81), (**B**) in the male population (*n* = 43, blue line) and in the female population (*n* = 38, red line) in the differentiation between groups MS+ (metabolic syndrome) and MS− (without metabolic syndrome).

The mean value (±standard deviation) of the receiver operating characteristic (ROC) curve for vaspin in the male population was higher (0.784 ± 0.094, *p* = 0.013) than in the female population (0.416 ± 0.115; for details, see Figure 4 and Table 9). Only the male subgroup demonstrated significant discriminative performance, whereas no significant discriminatory ability was observed in the overall study population or in women (Figure 4 and Table 9).

## 4. Discussion

In the present study, we found that the associations between circulating vaspin concentrations and metabolic parameters were sex-dependent. In men, higher serum vaspin concentrations were observed in subjects with metabolic syndrome as well as in individuals with more pronounced insulin resistance and hyperinsulinaemia. Moreover, serum vaspin concentrations were positively correlated with BMI, waist circumference, body fat percentage, fasting insulin, HOMA-IR, and diastolic blood pressure. In contrast, these associations were less pronounced in women, in whom the only statistically significant finding was a weak negative correlation between vaspin concentration and waist circumference. Importantly, although statistically significant, the observed associations were of moderate strength and should therefore be interpreted with caution. These findings suggest that sex-specific differences in vaspin regulation may be associated with the severity of metabolic disturbances related to visceral obesity.

Vaspin (visceral adipose tissue-derived serine protease inhibitor; SERPINA12) belongs to the serpin family of serine protease inhibitors. It was first isolated from visceral adipose tissue of OLETF (Otsuka Long-Evans Tokushima Fatty) rats, an experimental model of abdominal obesity accompanied by type 2 diabetes mellitus, insulin resistance, hypertension, and dyslipidaemia, which reflects the human metabolic syndrome phenotype [1].

Recent experimental studies have additionally suggested that vaspin may function as a batokine involved in the regulation of brown adipose tissue (BAT) activity. Vaspin has been shown to interact with low-density lipoprotein receptors (LRP1, LDLR, and VLDLR), thereby inhibiting adrenergic signalling and lipolysis in both brown and white adipocytes. These effects are mediated through the modulation of phosphodiesterase activity and endocytic lipid uptake [26].

Women exhibit higher UCP1 (uncoupling protein 1) expression and greater BAT activity than men, largely owing to estrogen-dependent regulation of mitochondrial function and sympathetic activity, which enhances their thermogenic capacity. Animal studies have demonstrated that vaspin suppresses thermogenesis and lipolysis, which may represent an adaptive mechanism protecting against excessive energy expenditure under conditions of metabolic dysfunction [27].

Kocabas and Sanlier suggested that elevated circulating vaspin concentrations observed in obesity and insulin resistance may represent an adaptive response aimed at limiting metabolic disturbances and maintaining energy homeostasis [28].

The results of previous studies investigating the relationship between circulating vaspin concentrations and obesity remain inconsistent [29]. Most studies have reported increased vaspin gene expression and elevated circulating concentrations of this adipokine with increasing BMI, body fat percentage, and visceral adipose tissue accumulation [12,13,14,30,31,32,33]. Our findings in men are consistent with these observations, as serum vaspin concentrations were positively correlated with BMI, waist circumference, and body fat percentage. In addition, vaspin concentrations were negatively correlated with the percentage of lean body mass.

However, contradictory findings have also been reported. For example, von Loeffelholz et al. observed the highest circulating vaspin concentrations in individuals with normal body weight compared with underweight, overweight, and obese subjects [32], whereas Körner et al. reported lower vaspin concentrations in obese girls than in their lean counterparts [33]. In contrast, Yin et al. found no association between circulating vaspin concentrations and anthropometric parameters in either individuals with normal glucose tolerance or patients with diabetes mellitus [34].

These discrepancies may result from differences in study populations, age, severity of metabolic disturbances, adipose tissue distribution, pharmacological treatment, and hormonal status. Serum vaspin concentrations may be more strongly associated with visceral adipose tissue accumulation and metabolic dysfunction than with BMI alone. Contemporary concepts of obesity pathophysiology emphasise that the development of metabolic disorders depends not only on adipose tissue quantity but primarily on its metabolic dysfunction, including chronic low-grade inflammation, impaired adipokine secretion, and reduced lipid storage capacity [35].

Experimental studies suggest that vaspin may exert insulin-sensitising and glucose-lowering effects [36,37]. Administration of recombinant vaspin to obese mice fed a high-fat diet improved glucose tolerance, enhanced insulin sensitivity, and reduced food intake [38]. The antidiabetic effects of this adipokine are believed to be associated with inhibition of insulin degradation induced by human kallikrein 7 (hK7), as insulin has been identified as a substrate of this protease. Furthermore, vaspin may modulate insulin signalling pathways, including PI3K/AKT-related mechanisms, and reduce inflammatory processes by suppressing the secretion of pro-inflammatory adipokines [1,39,40].

The positive associations observed between circulating vaspin concentrations, fasting insulin levels, and HOMA-IR in our study are consistent with the findings of El-Mesallamy et al., who demonstrated elevated vaspin concentrations in both lean and obese patients with type 2 diabetes mellitus compared with healthy individuals [41], and with those of Klöting et al., who reported higher vaspin gene expression in the visceral adipose tissue of patients with type 2 diabetes than in subjects with normal glucose tolerance [42]. Additionally, Li et al. demonstrated that vaspin gene expression in adipocytes from subjects with normal glucose tolerance increased together with body fat percentage and BMI [43]. Importantly, Hida et al. reported that vaspin gene expression may decline with the progression of diabetes mellitus [6], suggesting that compensatory upregulation of this adipokine may be transient and diminish as metabolic dysfunction advances.

Previous studies also suggest a potential role of vaspin in blood pressure regulation, atherosclerosis, and cardiovascular disease. In our study, circulating vaspin concentrations were positively associated with diastolic blood pressure in men. Moreover, multivariable regression analysis demonstrated that, after adjustment for fasting insulin concentration and lean body mass percentage, each 1 mmHg increase in diastolic blood pressure was associated with a 19 pg/mL increase in circulating vaspin concentration. No such association was observed in women. This finding is partially consistent with the observations of Esteghamati et al., who demonstrated positive correlations between vaspin concentrations and blood pressure in men with metabolic syndrome [44], as well as with those of Serinkan Cinemre et al., who reported significantly lower vaspin levels in hypertensive patients compared with normotensive individuals [45].

Recent evidence suggests that vaspin may play a role in endothelial function and blood pressure regulation. It has been suggested that vaspin increases nitric oxide bioavailability, reduces oxidative stress and vascular inflammation, and may inhibit vascular remodelling associated with obesity and metabolic syndrome. Vaspin has also been proposed as a potential biomarker of cardiovascular risk in patients with metabolic disturbances [11,39,40]. Breitfeld et al. showed that intraperitoneal administration of human recombinant vaspin to female leptin-deficient obese db/db mice for four weeks significantly reduced blood triglyceride levels [10]. Furthermore, Kobat et al. demonstrated lower circulating levels of this molecule in patients with coronary artery disease compared with healthy controls [46], whereas Kadoglou et al. observed lower vaspin concentrations in patients after ischaemic stroke [47], which may support a protective role of vaspin in vascular dysfunction. Therefore, elevated vaspin concentrations observed in hypertension may reflect a compensatory response to endothelial dysfunction and vascular injury.

The observed positive correlation between vaspin and TAS levels in the MS+ group may also suggest a protective role of this adipokine. Animal studies have shown that vaspin administration suppresses reactive oxygen species generation during inflammatory activation of pulmonary vascular endothelial cells, at least partially via activation of the Akt/protein kinase B and glycogen synthase kinase-3β signaling pathway [48]. The human body is continuously exposed to free radicals (mainly oxygen-derived), which attack cellular and intracellular structures. Silvestrini and Mancini pointed out that in men with metabolic syndrome, plasma TAS levels may be artificially elevated because high levels of uric acid characterize this group compared to women (i.e., a substance with strong antioxidant potential in plasma but a pro-oxidant effect in tissues) [49]. Oxidant–antioxidant interactions in individuals with metabolic syndrome are highly dynamic. In our previous research, following a 28-day exercise programme, plasma TAS increased only in the MS− women group (compared with the first day of the study), while the MS+ women group mainly showed reduced fat mass without a significant change in TAS, suggesting a blunted antioxidant adaptation [4].

Imbalances within the oxidative–antioxidative system are regarded as key contributors to the development of many disorders, including those related to glucose metabolism, insulin regulation, lipid homeostasis, and endothelial function. Chronic hyperglycemia and dyslipidemia enhance mitochondrial production of reactive oxygen species (ROS) and stimulate enzymatic ROS-generating pathways, such as nicotinamide adenine dinucleotide phosphate (NADPH) oxidase, while antioxidant defense mechanisms include superoxide dismutase (SOD), catalase (CAT), and glutathione peroxidase (GPx), together with non-enzymatic, low-molecular-weight antioxidants such as reduced glutathione (GSH), uric acid, bilirubin, ascorbic acid, and α-tocopherol present in plasma. When ROS formation exceeds endogenous antioxidant defenses, oxidative stress-related complications may develop [50]. Because TAS captures the combined redox-buffering capacity of these systems, it provides a more comprehensive assessment of antioxidant potential than individual markers measured separately. In patients with metabolic syndrome, TAS may be decreased (indicating antioxidant depletion) or paradoxically increased as a compensatory response to chronic oxidative stress.

Interestingly, the pattern of associations between vaspin concentrations and anthropometric parameters differed between women and men. In men, higher vaspin concentrations were positively associated with BMI, waist circumference, and body fat percentage, and negatively associated with lean body percentage. ROC curve analysis showed that circulating vaspin concentrations discriminated moderately between participants with and without metabolic syndrome in men (AUC = 0.784), whereas no significant discriminative ability was observed in women. These findings further support the concept that circulating vaspin concentrations are more closely associated with metabolic disturbances in men than in women. In contrast, women showed an opposite direction of these associations, with lower vaspin concentrations observed with increasing waist circumference, together with similar trends for WHR and body fat percentage. Although these findings should be interpreted cautiously due to the limited sample size and the lack of detailed hormonal information, they may suggest sex-specific differences in the regulation of vaspin secretion. Potential explanations may include differences in adipose tissue distribution, insulin sensitivity, and hormonal regulation, particularly the influence of menopausal status and sex hormones. Further studies with larger cohorts and comprehensive hormonal assessment are needed to clarify these sex-dependent patterns.

Previous studies investigating sex-related differences in vaspin concentrations have been inconclusive. Some studies demonstrated higher serum vaspin concentrations in women than in men, whereas others failed to confirm such differences. Danuaji et al. reported significantly higher serum vaspin concentrations in women with polycystic ovary syndrome, regardless of BMI [51].

Körner et al. suggested that sex-related differences emerge primarily after puberty [33]. The less pronounced associations observed in women in our study may be related to greater hormonal heterogeneity within the female study population. The female group included both premenopausal and postmenopausal women, while we did not analyse the effects of hormonal status, hormone replacement therapy, and hormonal contraception.

In recent years, increasing attention has been paid to sexual dimorphism in adipose tissue metabolism and adipokine secretion. Men and women differ not only in adipose tissue distribution but also in adipocyte metabolic activity, adipose tissue inflammatory profile, and susceptibility to insulin resistance. Differences in the proportions of visceral and subcutaneous adipose tissue may substantially influence adipokine secretion and their metabolic significance. Visceral adipose tissue, which is generally more abundant in men, exhibits greater metabolic and pro-inflammatory activity than subcutaneous adipose tissue and is more strongly associated with insulin resistance, endothelial dysfunction, and cardiovascular complications [18,52,53,54,55]. This may partially explain why the associations between vaspin concentrations and metabolic parameters observed in our study were present predominantly in men and suggests that circulating vaspin concentrations may be more closely associated with visceral obesity-related metabolic dysfunction than with general adiposity assessed by BMI alone.

Based on our findings and previous experimental evidence, it may be hypothesized that elevated vaspin concentrations in men with more advanced metabolic disturbances could reflect an adaptive response to insulin resistance and chronic inflammation associated with visceral obesity. Increased secretion of this molecule may reflect a compensatory response that could potentially contribute to improved insulin sensitivity and protection against endothelial dysfunction. A similar compensatory increase has also been described for other potentially protective adipokines, such as adiponectin and omentin [56,57]. However, as metabolic disturbances progress and adaptive mechanisms are exhausted, this response may gradually weaken or become insufficient (for example, due to reduced receptor sensitivity). One possible hypothesis is that the coexistence of elevated circulating vaspin concentrations and persistent metabolic disturbances may reflect a phenomenon analogous to leptin or ghrelin resistance, tentatively referred to as “vaspin resistance” [58,59,60]. However, this hypothesis remains speculative and requires confirmation in future studies.

According to this hypothesis, elevated circulating vaspin concentrations observed in some obese individuals may reflect a compensatory response to reduced tissue sensitivity to this protein. This could partially explain the coexistence of elevated vaspin concentrations and persistent metabolic disturbances despite the potentially beneficial effects of this adipokine on insulin sensitivity and endothelial function. Moreover, the concept of vaspin resistance may help explain inconsistencies across clinical studies and the lack of unequivocal associations between circulating vaspin concentrations and obesity severity. Nevertheless, the lack of large prospective studies currently precludes definitive conclusions on whether elevated vaspin concentrations confer protective effects or merely reflect the severity of metabolic dysfunction [22,26,60].

### Limitations

The limitations of the present study include the relatively small sample size and cross-sectional design, which preclude causal inference. Although the study and control groups were recruited using different strategies, all participants were evaluated according to the same standardized study protocol. Nevertheless, the possibility of selection bias cannot be entirely excluded.

The lack of information regarding menopausal status, hormone replacement therapy, and hormonal contraception limited the assessment of their potential influence on sex-specific vaspin regulation. Therefore, the observed differences in the patterns of associations between vaspin concentrations and anthropometric parameters in women and men should be interpreted with caution.

Although waist circumference is a widely accepted surrogate marker of visceral adiposity and is included in the diagnostic criteria for metabolic syndrome, the lack of imaging-based methods (e.g., CT, MRI, or DEXA) limited a more precise assessment of visceral fat distribution.

Additionally, inflammatory biomarkers were not measured in the present study. Therefore, the proposed anti-inflammatory role of vaspin is based on previous experimental and clinical evidence and could not be directly evaluated in our study population.

Some participants from both study groups were receiving antihypertensive and/or lipid-lowering therapies, which may have influenced circulating vaspin concentrations. Due to the heterogeneity of pharmacological treatment and the relatively small sample size, the potential confounding effects of these medications could not be fully assessed.

Finally, sex-stratified analyses may have reduced statistical power, particularly in the female subgroup. The single-center design and inclusion of only Caucasian participants may also limit the generalizability of our findings. Therefore, further studies involving larger and more diverse populations are required to confirm these observations.

## 5. Conclusions

The present study demonstrated that circulating vaspin concentrations were more strongly associated with metabolic disturbances in men than in women. Positive associations between serum vaspin concentrations and BMI, waist circumference, body fat percentage, fasting insulin, HOMA-IR, and diastolic blood pressure suggest that vaspin reflects obesity- and insulin resistance-related metabolic dysfunction.

Our findings further suggest that circulating vaspin concentrations may be more closely associated with visceral obesity-related metabolic dysfunction than with general adiposity assessed by BMI alone.

Further prospective studies involving larger populations and including inflammatory markers and imaging-based assessment of visceral adipose tissue are needed to clarify the biological role of vaspin, verify the hypothesis of “vaspin resistance,” and determine whether vaspin has clinical utility as a biomarker of cardiometabolic risk.

Although circulating vaspin concentrations show promise as a marker of visceral obesity-related metabolic dysfunction, particularly in men, their clinical utility for identifying individuals at increased cardiometabolic risk requires confirmation in larger prospective studies.

## Figures and Tables

**Table 1:** Characteristics of the study population.

Parameter	MS+ (*n* = 60)	MS− (*n* = 21)	*p*
Height [cm]	172.66 ± 9.88	170.286 ± 9.82	NS *
Body weight [kg]	111.11± 13.87	68.03± 25.56	<0.0001 **
BMI [kg/m^2^]	37.10 ± 2.64	23.23 ± 7.04	<0.0001 **
Waist circumference [cm]	115.51 ± 10.03	79.42 ± 17.72	<0.0001 **
WHR	0.96 ± 0.07	0.81 ± 0.09	<0.0001 **
Body fat [%]	42.73 ± 4.14	21.87 ± 9.02	0.0003 ***
Body lean [%]	57.23 ± 4.13	78.12 ± 8.99	0.0003 ***

MS+—metabolic syndrome group, MS−—without metabolic syndrome group, *n*—number of people; *p*—level of statistical significance (t-Student); BMI—Body Mass Index; WHR—Waist–Hip Ratio; * *p* value according to the ANOVA independent test to data, that satisfy the assumption of normality in Shapiro–Wilk test and the assumption of homogeneity of variances in Levine’s test; ** *p* value according to the Mann–Whitney U test applied to data that did not satisfy the assumption of normality in Shapiro–Wilk test; *** *p* value according to the Welch’s test in cases of data with unequal variances despite normal distribution; NS—non-significant differences.

**Table 2:** Comparison of selected anthropometric parameters in the two study groups—women (*n* = 38) and men (*n* = 43).

Parameter	Women (*n* = 38)	Men (*n* = 43)	*p*
Age [years]	45 (39; 54)	43 (39; 50)	NS *
Body weight [kg]	87.700 (65.425; 108.325)	105.2 (88.35; 128.75)	0.001 *
BMI [kg/m^2^]	31.15 (24.30; 41.65)	33.6 (27.00; 41.55)	NS **
Waist circumference [cm]	102.50 (79.75; 115.50)	112 (94.00; 128.00)	0.003 *
WHR	0.854 (0.792; 0.918)	0.992 (0.903; 1.059)	<0.0001 **
Body fat [%]	42.000 (27.875; 52.775)	35.800 (26.150; 44.400)	NS **
Body lean [%]	58.000 (47.225; 72.125)	64.200 (55.600; 73.850)	<0.0001 **

Values are presented as medians (25%; 75% percentiles), *n*—number of people; *p*—level of statistical significance; BMI—Body Mass Index; WHR—Waist–Hip Ratio; * *p* value according to the ANOVA independent test to data, that satisfy the assumption of normality in Shapiro–Wilk test and the assumption of homogeneity of variances in Levine’s test; ** *p* value according to the Mann–Whitney U test applied to data that did not satisfy the assumption of normality in Shapiro–Wilk test; NS—non-significant differences.

**Table 3:** Comparison of selected parameters of glucose and lipid metabolism, blood pressure, total antioxidant status (TAS) and vaspin concentration in the two study groups—women (*n* = 38) and men (*n* = 43).

Parameter	Women (*n* = 38)	Men (*n* = 43)	*p*
Fasting glucose [mmol/L]	5.00 (4.72; 5.56)	5.51 (5.19; 5.93)	0.011 **
Fasting insulin [µU/mL]	7.75 (5.82; 12.22)	10.90 (7.65; 15.45)	NS **
HOMA-IR	1.77 (1.16; 2.93)	2.70 (1.79; 3.93)	0.035 **
Total cholesterol [mmol/L]	5.67 (4.76; 6.50)	5.10 (4.47; 5.72)	0.020 *
LDL-C [mmol/L]	3.49 (2.80; 4.56)	3.05 (2.62; 3.82)	0.013 ***
HDL-C [mmol/L]	1.47 (1.18; 1.83)	1.26 (0.95; 1.43)	0.021 **
Triglycerides [mmol/L]	1.22 (0.90; 1.75)	1.79 (1.19; 2.82)	0.005 **
SBP [mmHg]	134 (114.0; 146.5)	140 (130.0; 150.0)	NS **
DBP [mmHg]	86 (78.2; 92.0)	90 (82.5; 94.0)	NS **
TAS [mmol/L]	1.156 (0.912; 1.430)	1.730 (1.271; 2.435)	NS **
Vaspin [ng/mL]	0.372 (0.212; 1.088)	0.447 (0.160; 0.973)	NS **

Values are presented as medians (25%; 75% percentiles), *n*—number of people; *p*—level of statistical significance; HOMA-IR—Homeostatic Model Assessment-Insulin Resistance; LDL-C—Low Density Lipoprotein Cholesterol; HDL-C—High Density Lipoprotein Cholesterol, SBP—systolic blood pressure; DBP—diastolic blood pressure; TAS—total antioxidant status; * *p* value according to the ANOVA independent test to data, that satisfy the assumption of normality in Shapiro–Wilk test and the assumption of homogeneity of variances in Levine’s test; ** *p* value according to the Mann–Whitney U test applied to data, that did not satisfy the assumption of normality in Shapiro–Wilk test; *** *p* value according to the Welch’s test in cases of data with unequal variances despite normal distribution; NS—non-significant differences.

**Table 4:** Comparison of selected parameters of glucose and lipid metabolism, blood pressure, total antioxidant status (TAS) and vaspin concentration in the male group (*n* = 43): MS+ (*n* = 32) and MS− (*n* = 11).

Parameter	MS+ (*n* = 32)	MS− (*n* = 11)	*p*
Fasting glucose [mmol/L]	5.65 (5.09; 6.05)	5.22 (5.02; 5.41)	0.025 **
Fasting insulin [µU/mL]	13.20 (9.20; 18.27)	5.30 (3.65; 6.55)	0.0001 **
HOMA-IR	3.49 (2.21; 4.25)	1.15 (0.79; 1.59)	0.0001 **
Total cholesterol [mmol/L]	5.04 (4.48; 5.65)	5.17 (4.60; 5.68)	NS *
LDL-C [mmol/L]	3.05 (2.68; 3.82)	2.82 (2.52; 3.59)	NS **
HDL-C [mmol/L]	1.07 (0.91; 1.31)	1.66 (1.36; 2.22)	0.002 ***
Triglycerides [mmol/L]	2.18 (1.55; 3.15)	0.87 (0.71; 1.04)	0.0001 **
SBP [mm Hg]	140 (130.0; 153.5)	132 (125.0; 142.0)	NS **
DBP [mm Hg]	90 (83.0; 96.0)	86 (82.5; 91.0)	NS **
TAS [mmol/L]	1.775 (1.280; 2.726)	1.527 (1.191; 1.964)	NS **
Vaspin [ng/mL]	0.560 (0.360; 1.031)	0.124 (0.057; 0.326)	0.004 **

Values are presented as medians (25%; 75% percentiles), MS+—metabolic syndrome group, MS−—without metabolic syndrome group; *n*—number of people; *p*—level of statistical significance; HOMA-IR—Homeostatic Model Assessment-Insulin Resistance; LDL-C—Low Density Lipoprotein Cholesterol; HDL-C—High Density Lipoprotein Cholesterol, SBP—systolic blood pressure; DBP—diastolic blood pressure; TAS—total antioxidant status; * *p* value according to the ANOVA independent test to data, that satisfy the assumption of normality in Shapiro–Wilk test and the assumption of homogeneity of variances in Levine’s test; ** *p* value according to the Mann–Whitney U test applied to data, that did not satisfy the assumption of normality in Shapiro–Wilk test; *** *p* value according to the Welch’s test in cases of data with unequal variances despite normal distribution; NS—non-significant differences.

**Table 5:** Comparison of selected parameters of glucose and lipid metabolism, blood pressure, total antioxidant status (TAS) and vaspin concentration in the female group (*n* = 38): MS+ (*n* = 28) and MS− (*n* = 10).

Parameter	MS+ (*n* = 28)	MS− (*n* = 10)	*p*
Fasting glucose [mmol/L]	5.37 (4.94; 5.88)	4.77 (4.68; 4.87)	0.0006 ***
Fasting insulin [µU/mL]	10.00 (7.07; 14.15)	4.40 (3.70; 6.22)	0.0005 **
HOMA-IR	2.46 (1.64; 3.59)	0.93 (0.80; 1.20)	0.0001 **
Total cholesterol [mmol/L]	6.15 (4.96; 6.58)	4.90 (4.66; 5.34)	NS *
LDL-C [mmol/L]	4.37 (3.24; 4.67)	2.83 (2.64; 3.30)	0.0003 ***
HDL-C [mmol/L]	1.22 (1.06; 1.63)	1.94 (1.84; 2.17)	0.0001 **
Triglycerides [mmol/L]	1.43 (1.15; 1.86)	0.76 (0.65; 0.86)	<0.0001 ***
SBP [mm Hg]	140 (132.7; 150.0)	105.5 (101.2; 111.5)	<0.0001 *
DBP [mm Hg]	90 (84.2; 95.2)	71.5 (70.2; 76.5)	0.0001 **
TAS [mmol/L]	1.156 (0.945; 1.562	1.140 (0.931; 1.282)	NS *
Vaspin [ng/mL]	0.367 (0.203; 1.074)	0.475 (0.266; 1.748)	NS **

Values are presented as medians (25%; 75% percentiles), MS+—metabolic syndrome group, MS−—without metabolic syndrome group; *n*—number of people; *p*—level of statistical significance; HOMA-IR—Homeostatic Model Assessment-Insulin Resistance; LDL-C—Low Density Lipoprotein Cholesterol; HDL-C—High Density Lipoprotein Cholesterol, SBP—systolic blood pressure; DBP—diastolic blood pressure; TAS—total antioxidant status; * *p* value according to the ANOVA independent test to data, that satisfy the assumption of normality in Shapiro–Wilk test and the assumption of homogeneity of variances in Levine’s test; ** *p* value according to the Mann–Whitney U test applied to data, that did not satisfy the assumption of normality in Shapiro–Wilk test; *** *p* value according to the Welch’s test in cases of data with unequal variances despite normal distribution; NS—non-significant differences.

**Table 6:** Spearman’s rank correlation of vaspin concentrations versus selected anthropometric parameters in women (*n* = 38), men (*n* = 43) and the entire population (*n* = 81).

Parameter	Women (*n* = 38)		Men (*n* = 43)		Entire Population (*n* = 81)	
	R_s_	*p*	R_s_	*p*	R_s_	*p*
Body weight [kg]	−0.228	0.067	0.385	0.005	0.072	0.261
BMI [kg/m^2^]	−0.248	0.084	0.426	0.002	0.078	0.246
Waist circumference [cm]	−0.272	0.049	0.437	0.002	0.080	0.239
WHR	−0.262	0.057	0.317	0.017	0.064	0.284
Body fat [%]	−0.261	0.057	0.423	0.002	0.044	0.348
Body lean [%]	0.261	0.057	−0.423	0.002	0.043	0.352

R_s_—Spearman’s rank correlation coefficient for data that do not meet the assumption of normal distribution in the Shapiro–Wilk test or the assumption of homogeneity of variances in Levene’s test; *p*—level of statistical significance; BMI—Body Mass Index, WHR—Waist–Hip Ratio.

**Table 7:** Correlation of vaspin concentrations versus selected physiological and biochemical parameters in women (*n* = 38), men (*n* = 43) and the entire population (*n* = 81).

Parameter	Women (*n* = 38)		Men (*n* = 43)		Entire Population (*n* = 81)	
	R_s_	*p*	R_s_	*p*	R_s_	*p*
Age [years]	0.064	0.352	0.302	0.025	0.167	0.068
Fasting glucose [mmol/L]	−0.028	0.434	0.151	0.167	0.052	0.324
Fasting insulin [µU/mL]	−0.093	0.290	0.403	0.004	0.166	0.069
HOMA-IR	−0.091	0.294	0.429	0.002	0.168	0.066
Total cholesterol [mmol/L]	−0.162	0.165	0.033	0.417	−0.082	0.234
LDL-C [mmol/L]	−0.209	0.104	0.082	0.301	−0.078	0.245
HDL-C [mmol/L]	0.216	0.097	−0.200	0.099	−0.046	0.342
Triglycerides [mmol/L]	−0.069	0.341	0.243	0.058	0.134	0.117
SBP [mmHg]	−0.052	0.379	0.246	0.056	0.105	0.175
DBP [mmHg]	−0.111	0.254	0.329	0.016	0.115	0.153

R_s_—Spearman’s rank correlation coefficient used for variables that did not meet the assumptions of normal distribution in Shapiro–Wilk test and the assumption of homogeneity of variances in Levine’s test; *p*—level of statistical significance; HOMA-IR—Homeostatic Model Assessment of Insulin Resistance; LDL-C—Low-Density Lipoprotein Cholesterol; HDL-C—High-Density Lipoprotein Cholesterol, SBP—systolic blood pressure, DBP—diastolic blood pressure.

**Table 8:** Comparison of two age-adjusted regression models explaining the variation in serum vaspin concentrations before and after adding diastolic blood pressure to the basic model. The basic model included fasting insulin concentration and lean body percentage. Models are presented separately for men (**A**,**B**) and women (**C**,**D**). (**A**). Basic model (model includes two variables: fasting insulin and body lean [%]) in the male population (*n* = 43; R^2^_adj._ = 0.078; F = 2.186; *p*_(model)_ = 0.105). (**B**). Enlarged model (model includes three variables: fasting insulin, body lean [%] and diastolic blood pressure) in the male population (*n* = 43; R^2^_adj._ = 0.152; F = 2.880; *p*_(model)_ = 0.035). (**C**). Basic model (model includes two variables: fasting insulin and body lean [%]) in the female population (*n* = 38; R^2^_adj_. = 0.015; F = 1.0181; *p*_(model)_ = 0.397). (**D**). Enlarged model (model includes three variables: fasting insulin, body lean [%] and diastolic blood pressure) in the female population (*n* = 38; R^2^_adj_. = 0.007; F = 0.913; *p*_(model)_ = 0.468).

Predictor	b	SE	95% CI	*p*
A				
Body lean [%]	−0.021	0.012	−0.045–0.003	0.089
Insulin [µU/mL]	0.002	0.015	−0.028–0.032	0.886
B				
Predictor	b	SE	95% CI	*p*
Body lean [%]	−0.015	0.012	−0.040–0.009	0.201
Insulin [µU/mL]	0.001	0.014	−0.028–0.029	0.989
DBP [mmHg]	0.020	0.010	−0.001–0.040	0.043
C				
Predictor	b	SE	95% CI	*p*
Body lean [%]	0.014	0.013	−0.013–0.042	0.286
Insulin [µU/mL]	−0.010	0.027	−0.064–0.044	0.703
D				
Predictor	b	SE	95% CI	*p*
Body lean [%]	0.022	0.016	−0.011–0.055	0.190
Insulin [µU/mL]	−0.005	0.028	−0.061–0.051	0.854
DBP [mmHg]	0.013	0.016	−0.020–0.046	0.433

The regression model was adjusted for patients’ age; R^2^_adj_.—adjusted R-squared; F—F statistic; b—regression coefficient; SE—standard error; CI—confidence interval; *p*—level of statistical significance; DBP—diastolic blood pressure.

**Table 9:** Characteristics of receiver operating characteristic (ROC) curves for vaspin concentration in the entire group (*n* = 81), the in the male population (*n* = 43) and the female population (*n* = 38) for pairs of studied groups (MS+ vs. MS−).

Parameter	Sensitivity [%]	Specificity [%]	AUC	SD (AUC)	95% CI	*p*
Vaspin [ng/mL] in the entire group	56.5	57.1	0.612	0.081	0.454–0.770	0.127
Vaspin [ng/mL] in the male population	75.0	72.7	0.784	0.094	0.599–0.969	0.005
Vaspin [ng/mL] in the female population	57.1	60.0	0.416	0.115	0.190–0.642	0.436

MS+—metabolic syndrome group, MS−—without metabolic syndrome group; *n*—number of people; ROC—receiver operating characteristic, AUC—area under curve of receiver operating characteristic; SD (AUC)—standard deviation of AUC; CI—confidence interval; *p*—level of statistical significance.

## Data Availability

The data presented in this study are not publicly available due to privacy and ethical restrictions.

## Supplementary Materials

The following supporting information can be downloaded at: https://www.mdpi.com/article/10.3390/jcm15166284/s1. Table S1. Calculation formula for sensitivity, specificity and 95% CI of AUC (area under the receiver operating characteristic (ROC) curve) for metabolic syndrome (MS).

## Abbreviations

The following abbreviations are used in this manuscript:

BAT	Brown Adipose Tissue
BMI	Body Mass Index
WHR	Waist–Hip Ratio
WC	Waist Circumference
MS	Metabolic Syndrome
SBP	Systolic Blood Pressure
DBP	Diastolic Blood Pressure
HOMA-IR	Homeostatic Model Assessment-Insulin Resistance
LDL-C	Low-Density Lipoprotein Cholesterol
HDL-C	High-Density Lipoprotein Cholesterol
OLETF	Otsuka Long-Evans Tokushima Fatty
