# Supplementary material for: Beyond Obesity: Sex-Specific Associations of Vaspin with Metabolic Dysfunction
Source: J Clin Med. 2026 Aug 13;15(16):6284. doi: 10.3390/jcm15166284 (PMC13513227; doi:10.3390/jcm15166284)
Supplement: Supplementary file 1 [file jcm-15-06284-s001.zip › jcm-4458482-supplementary.pdf]

Supplementary

Table S1

Calculation formula for sensitivity, specificity and 95% CI of AUC (area under the receiver operating characteristic (ROC) curve) for metabolic syndrome (MS)

Parameters/Formula	MS Present	MS Absent
Index Test Positive	TP	FP
Index Test Negative	FN	TN
Sensitivity = TP / (TP + FN)		
Specificity = TN / (TN + FP)		
95% CI of AUC = AUC ± 1.96 SEM		

MS — metabolic syndrome; 95% CI of AUC—95% confidence interval in AUC (area under ROC curve) value; SEM—standard error of the mean; TP — true positive; FP — false positive; FN — false negative (FN); TN — true negative, according Managhan al. 2021.

Monaghan, T.F.; Rahman, S.N.; Agudelo, C.W.; Wein, A.J.; Lazar, J.M.; Everaert, K.; Dmochowski, R.R. Foundational statistical principles in medical research: Sensitivity, specificity, positive predictive value, and negative predictive value. Medicina 2021, *57*, 503.
